# Supplementary material for: An international validation of the Bolton Unistride Scale (BUSS) of tenacity
Source: PLoS One. 2022 Mar 11;17(3):e0264889. doi: 10.1371/journal.pone.0264889 (PMC8916625; doi:10.1371/journal.pone.0264889)
Supplement: Attachment — Submitted filename: rebuttal letter PLOS One 30.09.21.docx [file pone.0264889.s002.docx]

30^th^ September 2021

Dear Editors at PLOS One,

Our manuscript entitled “*An International Validation of the Bolton Unistride Scale (BUSS) of Academic Tenacity*” was previously submitted to PLOS One on 16.04.2021 (ref: PONE-D-21-07987). The submission did not reach review, but was returned to us asking to include a title page in the manuscript. For many reasons we were unable to respond in the required time and after 21 days our submission was withdrawn. As originally requested, we have now included a title page and are eager to re-submit our manuscript to PLOS One. In correspondence with Katy Jane Ruffell from the Editorial Office, we were advised to create a new submission and state the previously submitted manuscript reference number, which we have done. We believe that the manuscript is now suitable for consideration.

On behalf of all authors,

Rosie Allen
